# Crystal structure of Hop2–Mnd1 and mechanistic insights into its role in meiotic recombination

**DOI:** 10.1093/nar/gkv172

**Published:** 2015-03-03

**Authors:** Hyun-Ah Kang, Ho-Chul Shin, Alexandra-Styliani Kalantzi, Christopher P. Toseland, Hyun-Min Kim, Stephan Gruber, Matteo Dal Peraro, Byung-Ha Oh

**Affiliations:** 1 Department of Biological Sciences, KAIST Institute for the Biocentury, Cancer Metastasis Control Center, Korea Advanced Institute of Science and Technology, Daejeon 305–701, Korea; 2 Functional Genomics Research Center, Korea Research Institute of Bioscience and Biotechnology, Daejeon 305–806, Korea; 3 Laboratory for Biomolecular Modeling, Institute of Bioengineering, School of Life Sciences, École Polytechnique Fédérale de Lausanne (EPFL), and Swiss Institute of Bioinformatics (SIB), 1015 Lausanne, Switzerland; 4 Chromosome Organization and Dynamics, Max Planck Institute of Biochemistry, Am, Klopferspitz 18, 82152 Martinsried, Germany

## Abstract

In meiotic DNA recombination, the Hop2−Mnd1 complex promotes Dmc1-mediated single-stranded DNA (ssDNA) invasion into homologous chromosomes to form a synaptic complex by a yet-unclear mechanism. Here, the crystal structure of Hop2−Mnd1 reveals that it forms a curved rod-like structure consisting of three leucine zippers and two kinked junctions. One end of the rod is linked to two juxtaposed winged-helix domains, and the other end is capped by extra α-helices to form a helical bundle-like structure. Deletion analysis shows that the helical bundle-like structure is sufficient for interacting with the Dmc1-ssDNA nucleofilament, and molecular modeling suggests that the curved rod could be accommodated into the helical groove of the nucleofilament. Remarkably, the winged-helix domains are juxtaposed at fixed relative orientation, and their binding to DNA is likely to perturb the base pairing according to molecular simulations. These findings allow us to propose a model explaining how Hop2−Mnd1 juxtaposes Dmc1-bound ssDNA with distorted recipient double-stranded DNA and thus facilitates strand invasion.

## INTRODUCTION

In the meiotic cell cycle, homologous chromosomes interact with each other to form a synaptonemal complex (SC) characterized by a roughly parallel alignment of the homologous chromosome pairs (homologs) along their lengths (1). Genetic recombination during meiosis tends to occur between homologs rather than between non-homologs or sister chromatids. In most species, proteins required for SC formation are also required to promote inter-homolog crossing-over (2,3).

Meiotic recombination is initiated by the generation of DNA double strand breaks (DSBs), which is catalyzed by the evolutionary conserved protein Spo11 (4). When Spo11 forms DSBs, it remains covalently attached to the 5′ end of DNA (5,6). Endolytic cleavage by the Mre11–Rad50–Xrs2 or Mre11–Rad50–NBS1 complex and Sae2 in *S. cerevisiae* releases Spo11 attached to an oligonucleotide, as demonstrated in different organisms (7–9). The removal of Spo11-oligonucleotide is followed by bidirectional strand resection through the activity of Mre11 and Exo1 in *S. cerevisiae* and likely in mouse as well (10). The resulting single-stranded DNA (ssDNA) tails are bound by the RecA family member Rad51 or Dmc1, which forms a helical filamentous structure known as the presynaptic filament that is capable of invading into intact chromatids to form homologous joint molecules termed the synaptic complex (11–13). While Rad51 catalyzes recombination in both mitosis and meiosis, Dmc1 acts specifically in meiosis where Rad51 plays an accessory role (14,15). Unlike *E. coli* RecA, *S. cerevisiae* Rad51 requires protein cofactors, such as Rad54, for efficient D-loop formation (16).

Hop2 (for *ho*mologous *p*airing; also known as TBPIP) of *S. cerevisiae* is expressed specifically during meiosis. The *hop2*-null mutant of *S. cerevisiae* exhibits serious defects: synapsis between non-homologous chromosomes, unrepaired DNA double-strand breaks (DSBs) and arrests at the pachytene stage of meiosis (17). Mnd1 (for *m*eiotic *n*uclear *d*ivisions *1*) is also a meiosis-specific protein, and the *mnd1*-null mutation exhibits similar phenotypes as the *hop2*-null mutation (18). Later, Hop2 and Mnd1 were found to work together as a complex that promotes homologous chromosome pairing and DSB repair during meiosis (19). Consistent with the essential functions of the complex in meiosis, Hop2 and Mnd1 are found in many eukaryotic organisms, but not in *S. macrospora, D. melanogaster* and *C. elegans*, which also lack Dmc1 (20).

Biochemically, Hop2–Mnd1 was shown to physically interact with the Dmc1 or Rad51 nucleoprotein filament. It greatly promotes the Dmc1- and Rad51-mediated strand exchange between the presynaptic filament and recombining double-stranded DNA (dsDNA) to form a D-loop, suggesting that this activity of Hop2–Mnd1 may be responsible for the synaptic complex formation only between homologs during meiosis (21–26). Recently, Hop2–Mnd1 was shown to be broadly expressed in ALT (alternative lengthening of telomeres) cell lines and play a role in interchromosomal homology search in conjunction with Rad51 to drive telomere recombination in these mitotic cells (27).

Biochemical and mutagenic analyses revealed the presence of a DNA-binding winged-helix domain (WHD) at the N-terminus of both Hop2 and Mnd1 (26). Recently, a solution structure was determined for the WHD fragment of Hop2 (28). Combined small angle X-ray scattering (SAXS) and electron microscopic analyses showed that mouse Hop2–Mnd1 forms an elongated V-shaped molecule (26). The low resolution of the deduced molecular envelope, however, prevented correct interpretation of how the two molecules interact with each other.

In addition to Hop2–Mnd1, at least seven proteins or protein complexes are involved in the Dmc1-mediated DNA strand exchange step (29). As yet, their molecular mechanisms remain largely elusive. In order to provide structural information of the entire Hop2–Mnd1 complex at atomic resolution and to understand the mechanistic aspects of the Hop2–Mnd1 function, we have determined the crystal structure of full-length Hop2–Mnd1 derived from *Giardia lamblia*. The structure reveals that the WHDs of Hop2 and Mnd1 are closely juxtaposed via evolutionarily conserved interdomain interactions—thereby forming a joint dsDNA-interacting interface—and attached to an elongated, crescent-shaped Dmc1-binding structure. The ensuing analyses highlight structural features of Hop2–Mnd1 that appear critical for promoting the formation of synaptic complexes.

## MATERIALS AND METHODS

### Protein purification

The full-length DNA encoding for Hop2 (codon-optimized) and Mnd1 derived from *G. lamblia* ATCC 50803 were inserted into a modified pRSFDuet-1 vector (Novagen) by standard PCR-based cloning methods. Mnd1 with an N-terminal (His)_10_ tag and Hop2 without a tag were co-expressed from this vector in the *E. coli* BL21(DE3) RIPL strain (Novagen) at 18 ^o^C overnight. Cleared cell lysate was applied onto a gravity flow column containing HisPur^TM^ Cobalt Resin (Thermo Scientific). The column was washed with Buffer A (20 mM Tris-HCl pH 7.5, 0.1 M NaCl, 2 mM 2-mercaptoethanol), and the Hop2–Mnd1 complex was eluted with Buffer A containing additional 150 mM imidazole. After treatment of the tobacco etch virus nuclear inclusion a protease overnight the complex was further purified using a Hitrap Q anion exchange column (GE Healthcare) and HiLoad 26/60 Superdex 75 gel filtration column (GE Healthcare). Selenomethionine (SelMet)-substituted Hop2 and Mnd1 were produced in the *E. coli* B834 (DE3) methionine auxotroph (Novagen) and purified as described above. The truncated Hop2–Mnd1 variants, Hop2(70–231)–Mnd1(71–203) ( = ΔWHD), Hop2(115–231)–Mnd1(111–203) ( = LZ2+LZ3wCH) and Hop2(144/231)–Mnd1(140/203) ( = LZ3wCH), were constructed and cloned into the modified pRSFDuet-1 vector by standard cloning methods. These complexes, commonly containing a (His)_10_-tag on the Mnd1 fragment, were co-expressed in the *E. coli* BL21(DE3) RIPL strain, and purified as described above.

*Giardia lambila* Dmc1 was expressed from a modified version of the pMAL vector (New England BioLabs) in the *E. coli* pLysS (DE3) strain (Novagen) at 18 ^o^C overnight. The over-expressed N-terminal (His)_10_-MBP tagged Dmc1 was purified according to the same procedures described above except that buffer A containing 0.7 M NaCl was used for washing His-Pur cobalt resin.

### Crystallization and structure determination

The Hop2–Mnd1 complex was crystallized using the hanging-drop vapor diffusion technique at 18°C in a solution containing 18% PEG 3350, 0.1 M Tris-HCl pH 8.5 and 2% Tascimate (Hampton Research). The SelMet-labeled complex was crystallized under the same crystallization condition. X-ray diffraction data were collected at the beamline 5C at the Pohang Accelerator Laboratory. A single-wavelength anomalous dispersion (SAD) data set was collected with a SelMet-substituted Hop2–Mnd1 crystal at the Se absorption peak. All diffraction data were processed with HKL2000 (30), and the data set was used for phase determination/improvement with SOLVE/RESOLVE (31). The model building and structure refinement were carried out against a native data set using the programs *COOT* (32) and *CNS* (33). Crystallographic data statistics are summarized in Table 1.

**Table 1.** Data collection and structure refinement statistics

Data collection	Native	Se-Met substituted
X-ray source^a^	BL5C, PAL	BL5C, PAL
Space group	*C*2	*C*2
Unit cell dimensions		
a, b, c (Å)	117.84, 69.06, 292.09	117.85, 69.07, 292.22
α, β, γ (°)	90, 95.35, 90	90, 95.34, 90
Wavelength (Å)	0.97889	0.97889
Resolution (Å)	50.0–3.2	50.0–3.5
*R*_sym_ (%)	7.4 (27.2)^b^	9.0 (21.9)
*I*/σ(*I*)	22.1 (3.1)	32.7 (7.9)
Completeness (%)	87.0 (64.9)	75.2 (54.7)
Redundancy	3.8 (1.9)	2.6 (1.6)
Figure of Merit		0.31
**Refinement**		
Resolution (Å)	50.0–3.2	
No. of reflections	34,750	
*R*_work_ / *R*_free_ (%)	23.9/28.6	
R.m.s deviations		
bond lengths (Å) / angles (°)	0.011 / 1.340	
Average B-values (Å^2^)	72.08	
Ramachandran plot (%)		
Most favored/Favored	95.5/4.4	
Generously allowed	0.1	

^a^Beamline 17A at Photon Factory.

^b^The numbers in parentheses are the statistics from the highest resolution shell.

### DNA sequences

The sequence of the 60-mer ssDNA used for the Exonuclease I protection assay was 5′- CGG CAT CAG AGC AGA TTG TAC TGA GAG TGC ACC ATA TGC GGT GTG AAA TAC CGC ACA GAT-3′. The 40 base-pair (bp) DNA used for the fluorescence anisotropy titration was prepared by annealing 5′- GCG GGT AAT CCA GAT GTT CCA CGT GAA ACA GAA CAA CTA A-3′ and 5′- TTA GTT GTT CTG TTT CAC GTG GAA CAT CTG GAT TAC CCG C-3′. The sequence of the 40-mer ssDNA used for the fluorescence anisotropy titration was 5′- GCG GGT AAT CCA GAT GTT CCA CGT GAA ACA GAA CAA CTA A-3′.

### Exonuclease I protection assay

Protection assays with *E. coli* exonuclease I was performed as reported (21,26). In a total of 10 μl reaction volume, 60-mer ssDNA (24 μM) was mixed with Dmc1 (12 μM) in a buffer B (50 mM Tris-HCl pH 7.5, 70 mM NaCl, 2 mM MgCl_2_, 2 mM ATP) and incubated for 10 min at 37 ^o^C. The Hop2–Mnd1 complex (2.5 μM) was added and incubated for 10 min followed by the addition of 10 units of exonuclease I (Thermo Scientific, 20 units/μl). After 20 min, the reaction was stopped by adding proteinase K (1 mg/ml) and further incubated for 20 min. The reaction mixtures were analyzed by electrophoresis on a 15% native acrylamide gel and ethidium bromide staining.

### Anisotropy titration measurements

Fluorescence anisotropy titrations were performed in triplicate at 25°C using a BioTek Neo plate reader with Hop2–Mnd1 and 5′-fluorescein-labeled 40 bp dsDNA or 40-mer ssDNA (50 nM) in a buffer containing 20 mM Tris-HCl (pH 7.5), 100 mM NaCl and 5 mM MgCl_2_. The dissociation constant was derived from the equations;

\begin{equation*} \begin{array}{*{20}l} {A_t = } \\ {\frac{{A_{DNA} \left( {[DNA]_t - [Hop2.DNA]} \right) + A_{Hop2.DNA} Q[Hop2.DNA]}}{{[DNA]_t - [Hop2.DNA] + Q[Hop2.DNA]}}} \\ \end{array} \end{equation*}

where *A*_t_ is the total anisotropy and

\begin{equation*} \begin{array}{*{20}l} {[Hop2.DNA] = } \\ {\frac{{\left( {[Hop2]_t + [DNA]_t + K_d } \right) - \sqrt {\left( {[Hop2]_t + [DNA]_t + K_d } \right)^2 - 4[Hop2]_t [DNA]_t } }}{2}} \\ \end{array} \end{equation*}

[Hop2]_t_ and [DNA]_t_ are the total concentrations of Hop2–Mnd1 and DNA. [Hop2.DNA] is the concentration of the Hop2–Mnd1:DNA complex. Q is the fluorescence intensity of Hop2–Mnd1:DNA relative to DNA.

### Molecular modeling and MD simulations

The structure of TtgV (PDB entry: 2xro) was superimposed onto each of the WHDs of Hop2–Mnd1 with the UCSF Chimera visualization program (v1.5.3) (34). The resulting two dsDNA segments were then connected to form a single dsDNA using the 3DNA software (v2.0) (35,36) to refine DNA backbone and base pairing geometry. In a final step, local geometry optimization was performed with the NAMD program (v2.8) (37) to refine DNA backbone and base pairing geometry. Next, the Hop2–Mnd1:dsDNA model was placed in an explicit solvent box with 0.15 M NaCl concentration under periodic boundary conditions. The complex was parameterized using the AMBER force field (ff99bsc0) and the TIP3P model was used for water molecules (LeaP program) (38). Geometry optimization via energy minimization and MD simulations were performed using the NAMD program (v2.8) (37). Initially, an energy minimization was performed for 10 000 steps, followed by an equilibration phase where the protein and nucleic acid atoms were gradually unrestrained and temperature was gently raised from 10 K to 310 K in 200 ps. Constant temperature of 310 K was enforced using Langevin dynamics and constant pressure of 1 atm was enforced through the Langevin piston during the production phase. In all phases a time step of 1 fs was used, the covalent bonds involving hydrogen atoms were constrained by the RATTLE algorithm and the Van der Waals interaction cutoff distances was set at 12 Å.

## RESULTS

### Overall structure

We attempted crystallization of human Hop2–Mnd1 without a success, and subsequently sought to crystallize remotely related homologs. In the public databases, two *G. lamblia* proteins GL50803_17044 and GL50803_6626 are annotated as a hypothetical protein and Mnd1, respectively (39). They are highly homologous to human Hop2 or Mnd1, since GL50803_17044 exhibits 24% sequence identity (49% similarity) with human Hop2 and GL50803_6626 exhibits 35% sequence identity (57% similarity) with human Mnd1. When coexpressed in *E. coli*, the two proteins formed a stable heterodimer, strongly suggesting that they are the orthologs of Hop2 and Mnd1 in *G. lamblia*.

**Figure 1.**
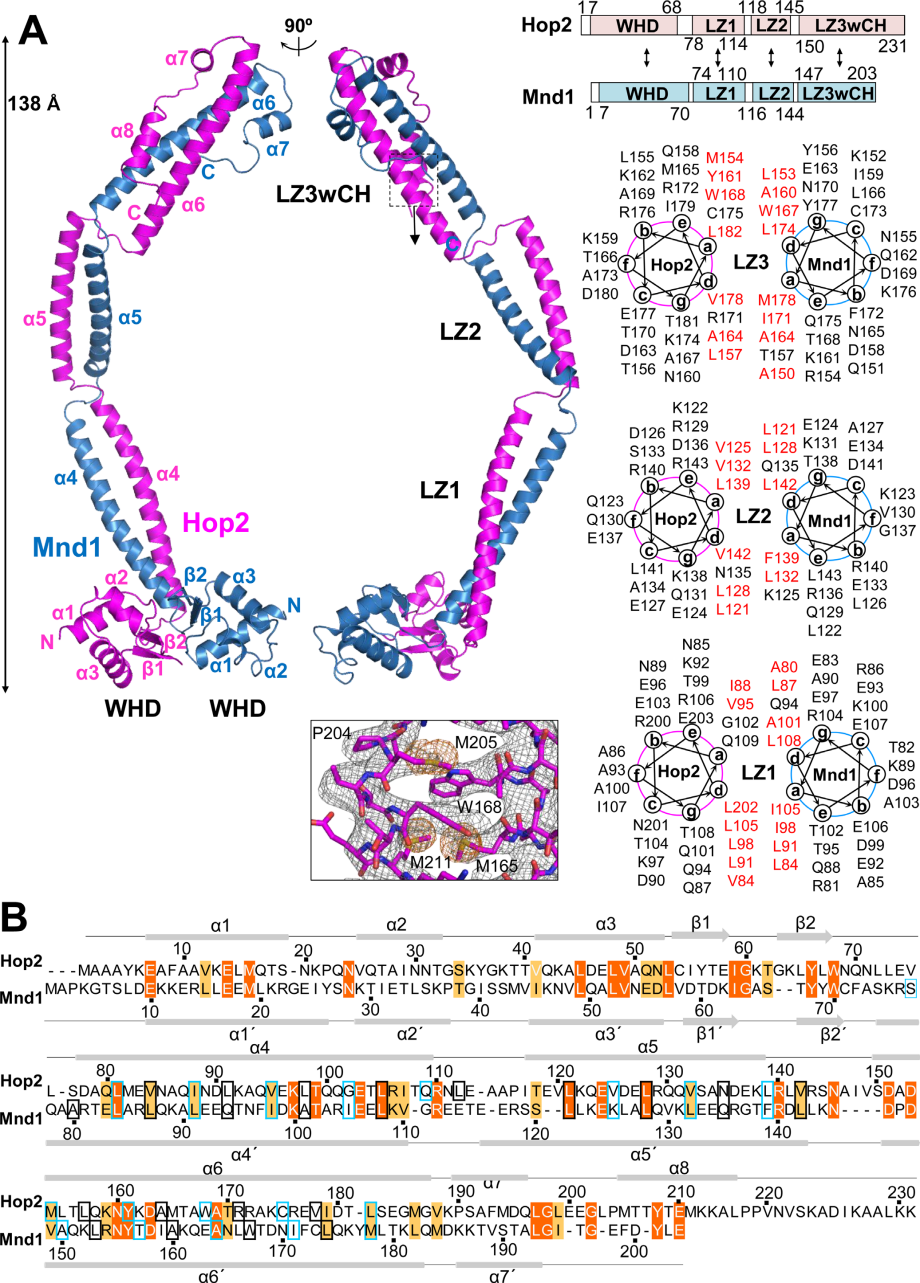
Overall structure of Hop2–Mnd1. (**A**) Two orthogonal views of the heterodimer. The two proteins are organized into an elongated complex with a parallel coiled coil and two WHDs at the N-terminal ends. Domain organizations (boxes) and intermolecular interactions (arrows) derived from the structure are shown schematically. Helical wheels of the three leucine zippers (LZ1, LZ2, LZ3) are shown. Hydrophobic residues at the *a* and *d* positions are in red. LZ3 is atypical in that it contains less leucine residues at the *d* positions. The experimental SAD map (contoured at 1σ) together with the final refined model is shown for the boxed region that contains selenomethione residues. (**B**) Structure-based sequence alignment of Hop2 and Mnd1. The two segments were aligned by Clustal X, and amino acid positions were adjusted according to the structural superposition of Hop2 and Mnd1. Two proteins exhibit 20% sequence identity. Identical residues are shown in orange and other conserved residues in apricot. Secondary structure elements are shown above (Hop2) and below (Mnd1) the alignment. Boxes indicate the positions *a* (sky blue) and *d* (black) in the heptad repeats.

The heterodimeric complex of full-length *G. lamblia* Hop2 (231 residues) and Mnd1 (203 residues) was crystallized subsequently. Nearly all the crystals exhibited very high anisotropic mosaicity, which was likely to arise from weak crystal packing interactions and intrinsic flexibility of the Hop2–Mnd1 heterodimer as described below. After extensive crystal screening to collect suitable X-ray data sets, the structure of Hop2–Mnd1 was determined by the single wavelength anomalous dispersion method using a selenomethionine-derivatized crystal (Table 1). A total of 14 methionine positions in the heterodimer facilitated chain tracing. The asymmetric unit of the crystal contained three copies of Hop2–Mnd1. Only one Hop2–Mnd1 heterodimer, referred to as Heterodimer I, exhibited electron densities for nearly the entire length of the molecules. This copy is used for the following structural description (Figure 1A).

The N-terminal ∼70 residues of both Hop2 and Mnd1 fold into winged-helix domains (WHDs) that are juxtaposed to each other through conserved interactions. The following 161 residues of Hop2 and 131 residues of Mnd1 both fold into three α-helices that are interrupted by two short non-helical regions. These α-helices of the two proteins together form a parallel coiled coil that provides the major interface for heterodimer formation. A heptad repeat analysis of the three coiled-coil helices exhibited the predominance of leucine at the *d* position, indicating that they are leucine zippers. We designate the most N-terminal leucine zipper as LZ1 and the following two as LZ2 and LZ3, respectively (Figure 1A). The non-helical regions form substantially kinked junctions between adjacent leucine zippers: the LZ1–LZ2 and LZ2–LZ3 junctions. Finally, the C-terminal segments of Hop2 and Mnd1 fold back onto the C-terminal leucine zipper (LZ3) to form a helical bundle-like structure, which is designated as LZ3wCH (for LZ3 with capping helices) (Figure 1A).

The combined length of the three α-helices forming the coiled coil is approximately 150 Å, and the height of the molecule including the WHDs is about 138 Å. With these dimensions, the overall structure of Hop2–Mnd1 is an elongated rod, which is curved due to the two kinked junctions. In reflection of the close structural similarity between Hop2 and Mnd1, the two proteins exhibit substantial sequence homology in a structure-based sequence alignment (Figure 1B).

### Intrinsic conformational flexibility

**Figure 2.**
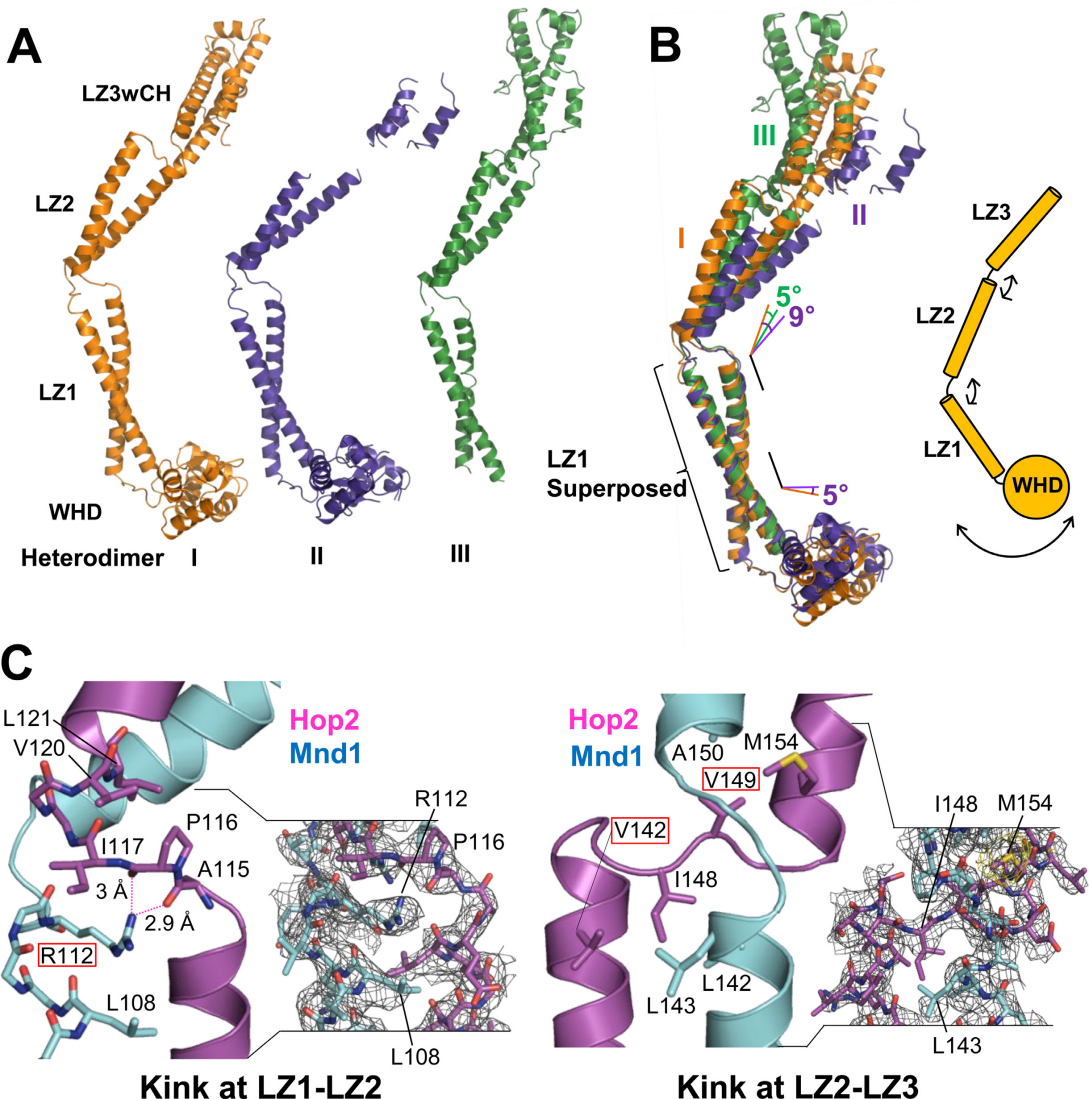
Conformational flexibility. (**A**) Three heterodimers in the asymmetric unit shown in the same orientation. Only Heterodimer I could be fully traced. (**B**) Structural superposition of Heterodimers I, II and III. Relative to LZ2 of Heterodimer I, LZ2s of Heterodimers II and III are displaced by 9° and 5°, respectively. The WHDs are displaced from one another by 5° between Heterodimers I and II, and that of Heterodimer III was invisible. (**C**) Detailed views of the kinked junctions between leucine zippers. Conserved residues are highlighted by rectangular boxes. Arg112 of Hop2 is sandwiched between Hop2 and Mnd1 residues. Dotted lines indicate hydrogen bonds. The experimental SAD map (contoured at 1σ) is shown for the two regions (two right panels).

Of the three heterodimers in the asymmetric unit, the electron densities for the WHD–LZ1 junction as well as the WHDs were visible in Heterodimers I and II. However, the electron densities for the WHD pair in Heterodimer III were not discerned from the noise level. Therefore, the WHD–LZ1 junction is presumed to be conformationally flexible, and the visibility of the WHDs in Heterodimers I and II is ascribed to the crystal packing interactions. Heterodimer II was mostly disordered beyond LZ2, as only a part of LZ3 could be traced (Figure 2A), indicating that the LZ2–LZ3 junction is not rigid. The LZ1–LZ2 junction also appears flexible according to structural superpositions of Heterodimers I and II or Heterodimers I and III, which show that the positions of LZ2 relative to LZ1 are different by about 9° and 5°, respectively (Figure 2B). The WHD–LZ1 junction appears most flexible, because the WHDs in Heterodimer III were indiscernible. The other two junctions may be less flexible, because all three heterodimers retain the curved rod shape (Figure 2A), and because similar molecular envelopes were observed by SAXS analysis of mouse Hop2–Mnd1 in solution (26). Presumably, the overall curved rod-like structure observed in the crystal is likely to be the inherent molecular shape of Hop2–Mnd1 under physiological conditions, while it is conformationally flexible at the three junctions to some degree.

### Junctions imparting curvatures

**Figure 3.**
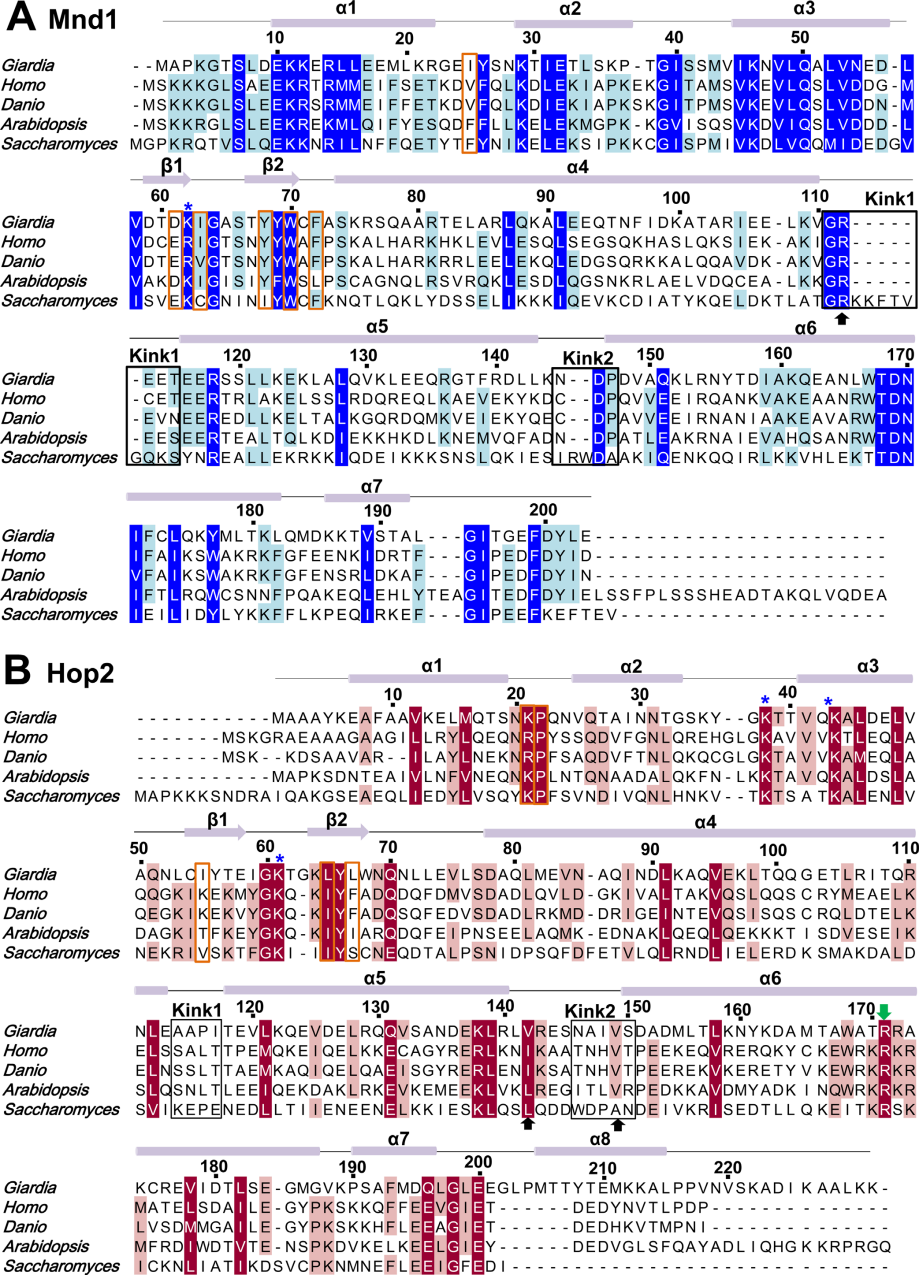
Multiple sequence alignments of Mnd1 and Hop2. (**A** and **B**) Homologs from the five different species are aligned: *Giarida lambila, Homo sapiens, Danio rerio, Arabidopsis thaliana* and *Saccharomyces cerevisiae*. The residues forming the kinked junctions are indicated by black rectangular boxes, and the black arrows indicate the residues highlighted in Figure 2C. The residues at the interface between WHDs are in orange boxes. The residues shown to be important experimentally for DNA binding or Dmc1 filament stabilization (26,28) are indicated by asterisks and a green arrow, respectively.

The LZ1–LZ2 junction, which introduces a ∼50° kink, involves Ala114-Ile117 of Hop2 and Gly111-T115 of Mnd1 both of which adopt a loop conformation. Arg112 of Mnd1 makes notable ionic interactions with two carbonyl oxygens of Ala115 and Pro116 of Hop2 (Figure 2C, left). In addition, Arg112 is mostly buried in between LZ1 and LZ2. Moreover, this residue is strictly conserved as a positively charged residue in five phylogenetically distant Mnd1 homologs (Figure 3A) and also in the top 100 homologs retrieved by a *BLAST* search. Pro116 of Hop2, which is conserved as a hydrophobic residue (Figure 3B), is involved in hydrophobic interactions with Val120 and Leu121 of Hop2. These interactions appear to be responsible for the junction segments to form the observed kink.

The LZ2–LZ3 junction, resulting in a ∼40° kink, involves Asn146-Ser150 of Hop2 and Asn145-Pro147 of Mnd1. The conformation of these segments appears to be mainly stabilized by Ile148 and Val149 of Hop2 that are involved in the hydrophobic interactions with the coil–coil interface residues (Figure 2C, right). While Ile148 of Hop2 is not a conserved residue, it is substituted by a hydrophobic residue or histidine whose side chain has a hydrophobic portion. Val149 of Hop2 is conserved or substituted by alanine (Figure 3B). Thus, the kink structures introducing curvatures to the Hop2–Mnd1 heterodimer is presumably an evolutionary conserved feature.

### Interaction with DNA

**Figure 4.**
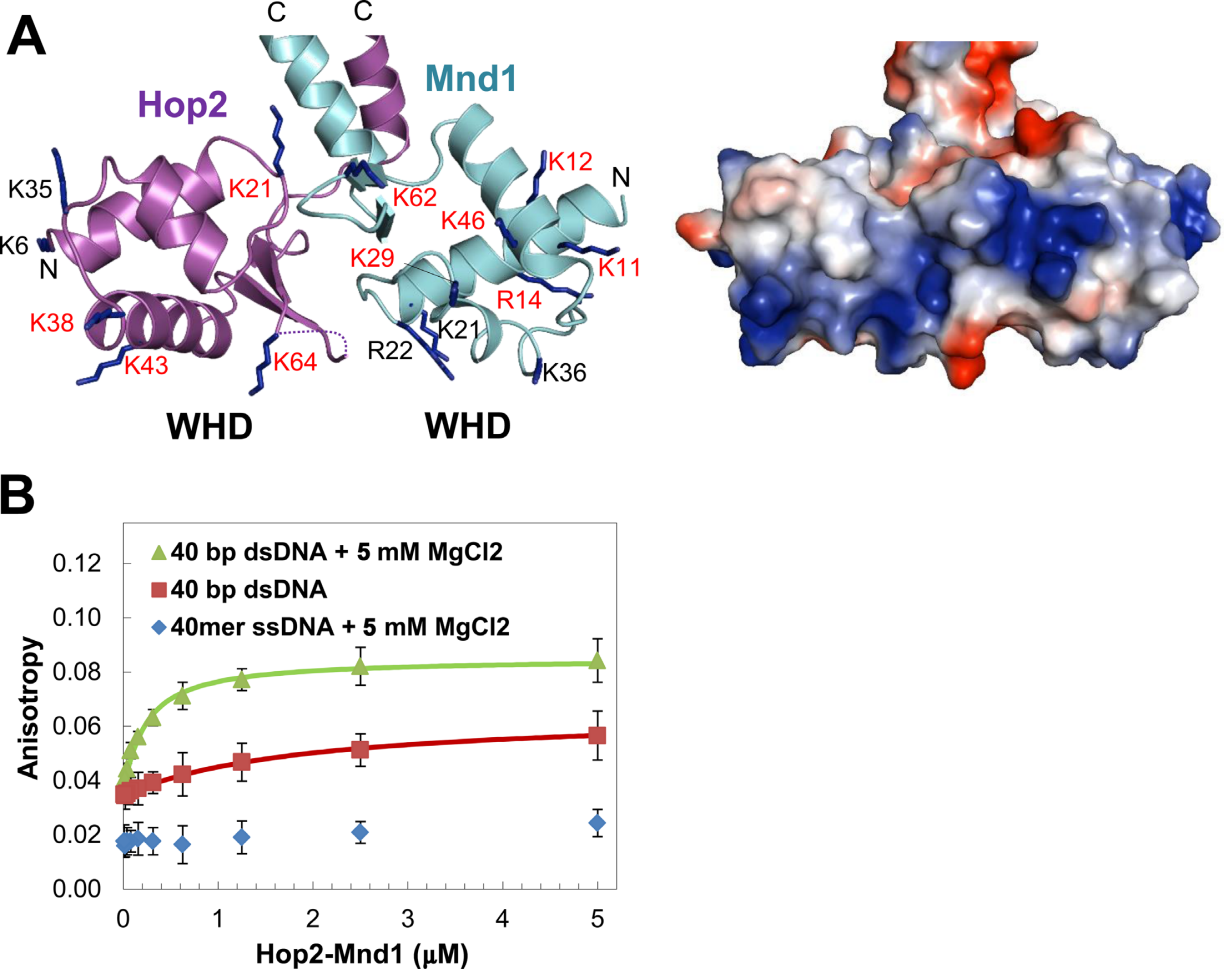
Interaction between the WHDs and DNA. (**A**) Basic patches across the WHDs. A ribbon drawing of the WHDs (left) and an electrostatic surface potential representation (right) are shown side by side. The basic residues are shown in sticks and conserved residues are labeled with red letters. Lys61 of Hop2 which is on the disordered wing is not included. (**B**) DNA binding of Hop2–Mnd1. Fluorescence anisotropy measurement was performed in triplicate by titrating Hop2–Mnd1 into fluorescein-labeled DNA (50 nM).

The dsDNA-binding activity of Hop2–Mnd1 has been well documented (21,22,24,25,40,41) and was recently attributed to the WHDs of the heterodimer (26,28). The WHDs of Hop2 and Mnd1 are structurally quite similar and composed of three-helix bundle with a C-terminal β-hairpin (wing). The WHD pair has a positively charged patch involving eleven conserved basic residues (Figures 3A, B and 4A), four of which were previously shown to be important for DNA binding by mutagenesis study (26,28). Consistently, *G. lambila* Hop2–Mnd1 interacted with 20 bp and 40 bp dsDNA in an electrophoretic mobility shift (not shown), as previously observed with mouse Hop2–Mnd1 (28,40). By fluorescence anisotropy titrations, we quantified the binding affinity of Hop2–Mnd1 for 40 bp dsDNA and 40-mer ssDNA. The apparent dissociation constant (*K*_D_) for 40 bp dsDNA measured by this method was 100 nM (Figure 4B). We noted that the presence of 5 mM MgCl_2_ enhanced the binding affinity for DNA by 10 times, which might explain an increased DNA condensation by Hop2–Mnd1 in the presence of divalent metal cations (31). Notably, the maximum fluorescence anisotropy values differ between the presence and the absence of Mg^2+^ (Figure 4B). The results indicate that Mg^2+^ is required for efficient binding to DNA and the absence of Mg^2+^ leads to poor DNA binding, and even a different DNA binding mode. The measured binding affinity is substantially higher than that of the mouse Hop2–Mnd1 for 20 bp dsDNA reported previously (*K*_D_ = 1.3 μM) (40). The discrepancy is attributed to the difference in the buffer conditions and the experimental methods. The ssDNA essentially failed to bind Hop2–Mnd1, as reported previously (24).

### Juxtaposed WHDs via conserved interdomain interactions

**Figure 5.**
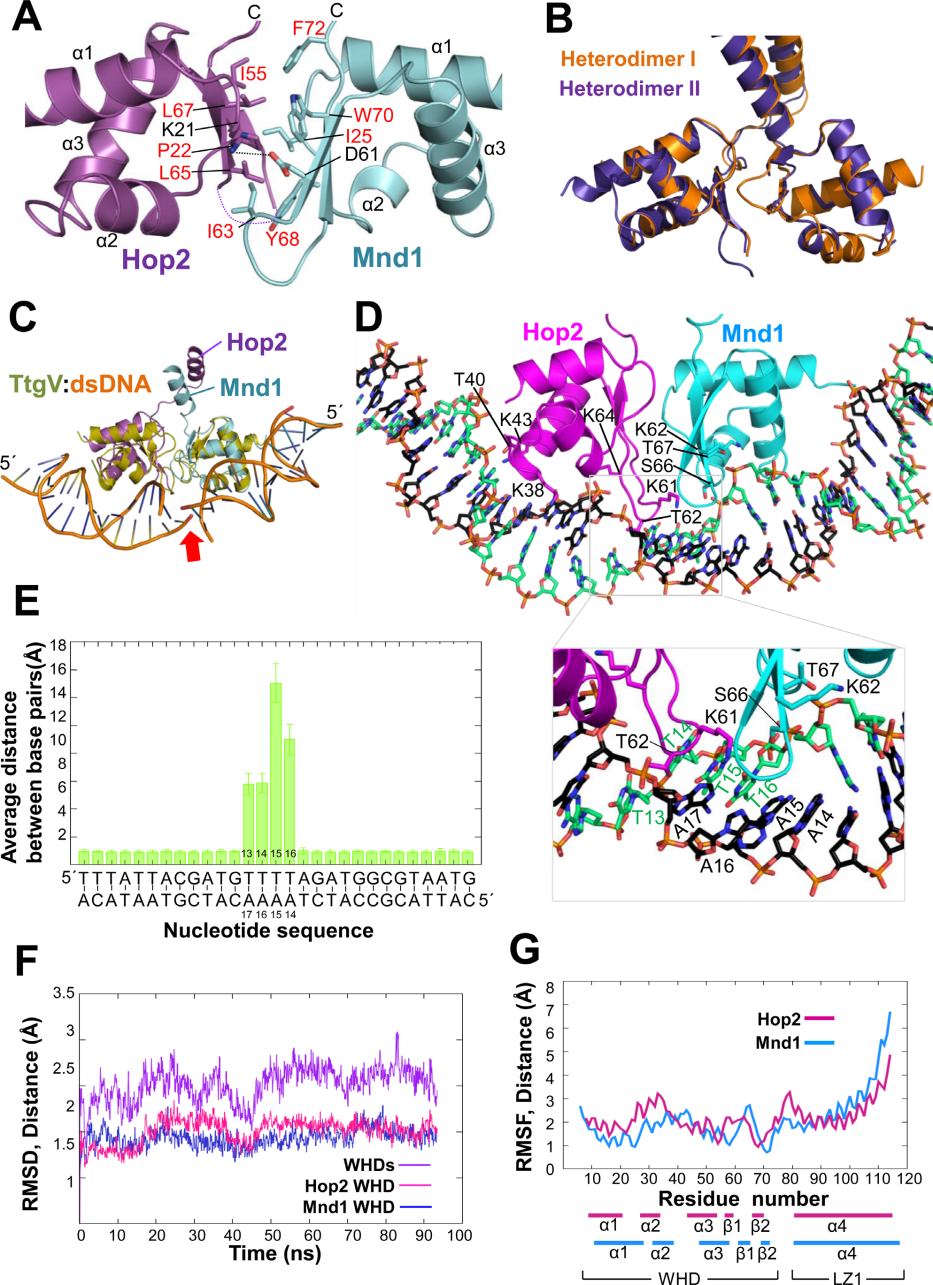
Juxtaposed WHDs and a model for dsDNA binding. (**A**) The interface between the WHDs is mostly hydrophobic. The residues at the interface are shown in sticks, with the hydrophobic residues labeled in red. The view is to look down from LZ1. (**B**) Superposition of the WHD pairs in Heterodimers I and II. The Cα traces were superposed only for Hop2. (**C**) Superposition of the WHD of TtgV bound to its recognition sequence (PDB entry: 2xro) onto each WHD of Hop2 and Mnd1. The arrow highlights the discontinuity of the DNA duplex. (**D**) A model for dsDNA binding to the WHD pair. The two dsDNA segments in *C* were connected to form a single dsDNA. The geometry was refined (see Methods section) and the resulting model is shown with the inset highlighting the observed dissociation of base pairs during ∼94 ns of MD simulation. Only the WHDs and LZ1 are included in the simulation. The view is to look down from LZ1. (**E**) MD-averaged base-pairing distances during ∼94 ns of MD simulation. Large deviations from canonical base-pairing geometries take place in the middle segment of dsDNA. (**F**) RMSDs between the crystal structure reference and each individual WHD and their heterodimer during the MD simulation time. RMSDs were calculated for all atoms. (**G**) RMSFs per residue during the simulation time. Much smaller fluctuation of the WHDs in comparison with LZ1 is noted. RMSFs were calculated for the Cα atoms only.

The two WHDs are closely juxtaposed and interact with each other (Figure 5A). The WHD–WHD interface, burying a surface area of 250.6 Å^2^, comprises of many hydrophobic residues (Pro22, Ile55, Leu65, Leu67 of Hop2; Ile25, Ile63, Tyr68, Trp70, Phe72 of Mnd1) and two charged residues (Lys21 of Hop2; Asp61 of Mnd1) forming a salt bridge (Figure 5A). These observations indicate that the WHDs adopt a fixed, rather than random, relative orientation. Consistent with this notion, the WHDs in Heterodimers I and II exhibit virtually the same orientations (Figure 5B). Remarkably, all of the interface lining residues are highly conserved except Ile55 of Hop2 (Figure 3A and B; orange boxes), indicating that the juxtaposition of the WHDs in the observed orientations is likely to be an evolutionary conserved feature important for the molecular function of Hop2–Mnd1. A further confirmation of the stability of the WHD–WHD interface comes from molecular dynamics (MD) simulations, as described in the next section.

### A model for dsDNA binding by the WHD pair

A prominent consequence of the WHD juxtaposition is to distort bound DNA, as exemplified by the LexA WHDs that causes overall 35° bending of DNA toward the major groove (42). So far, our effort to obtain crystals of Hop2–Mnd1 bound to dsDNA has been unsuccessful. Instead, we used available structural information to deduce how the WHD pair of Hop2–Mnd1 might bind dsDNA. The WHDs of both Hop2 and Mnd1 are structurally most similar to the WHD of transcription regulator TtgV (42) among the known structures of the WHDs in complex with DNA according to the program Dali (43). Structural superposition of the TtgV:dsDNA complex onto both WHDs of Hop2 and Mnd1 indicated that binding of the juxtaposed WHDs to a continuous DNA is likely to require severe distortion of the DNA (Figure 5C).

To investigate further, we modeled dsDNA bound to the WHDs based on the TtgV:dsDNA structure, and after geometry optimization, could confirm indeed that dsDNA is highly perturbed in the model. Based on this initial model, we performed MD simulations to further test the stability of the complex and the structural changes produced upon dsDNA binding. MD simulations revealed a distortion in the base pairing in between the WHDs (Figure 5D). In particular, the distances between the nucleotides pairs involved in binding increased well above 4 Å, producing a significant distortion of the canonical hydrogen-bonding pattern (Figure 5D and E). Although in the timescale explored by MD (i.e. ∼94 ns) a complete opening of the two strands was unobserved, this distortion could be an indication of an early stage of DNA melting. Since deviation from standard base-pairing requires energy, the observed DNA-binding mode could explain why triple-stranded DNA, which has forks in the middle of the molecule, binds Hop2–Mnd1 more tightly than dsDNA with similar length (22).

Consistent with the unspecific nature of Hop2–Mnd1 DNA binding, the most relevant interactions are observed with the phosphate backbone in MD simulations. A number of lysine residues, including Lys38, Lys43 and Lys61 of Hop2 and Lys62 of Mnd1 which were shown to be important for DNA binding (26,28), built the DNA-binding interface interacting with the phosphate backbone. Other frequent interactions were established by Thr40 of Hop2 (79% occupancy) and Thr67 of Mnd1 (64%). In addition to Thr67, Lys62 and Ser66, all on the β-hairpin of Mnd1, interacted with the DNA phosphate backbone. Similarly, Lys61, Lys64 and Thr62, all on the β-hairpin of Hop2, were observed to interact with the phosphate backbone. The combined action of these β-hairpins ( = wings) interacting with the minor groove might likely be responsible for the observed distortion in the base pairing. Overall, Hop2–Mnd1 residues appear to engage nucleotide bases unspecifically (average hydrogen-bonding occupancy < 20%).

In the MD simulation, the WHD–WHD interface was stable. In particular, all the interactions seen in the X-ray structure are maintained and the mutual orientation and distance of the WHD domains are well conserved. In particular, the center of mass distance of the WHDs is 23.4 ± 0.5 Å in MD compared with 23.3 Å observed in the crystal. Moreover, their root mean square deviations (RMSDs) from the crystallographic reference structure are quite low whether or not the WHDs interact with dsDNA, and their root mean square fluctuations (RMSFs) during the simulation time are low, further supporting the stability of the WHD–WHD interface (Figure 5F and G).

### Interaction between Hop2–Mnd1 and Dmc1 nucleofilament

**Figure 6.**
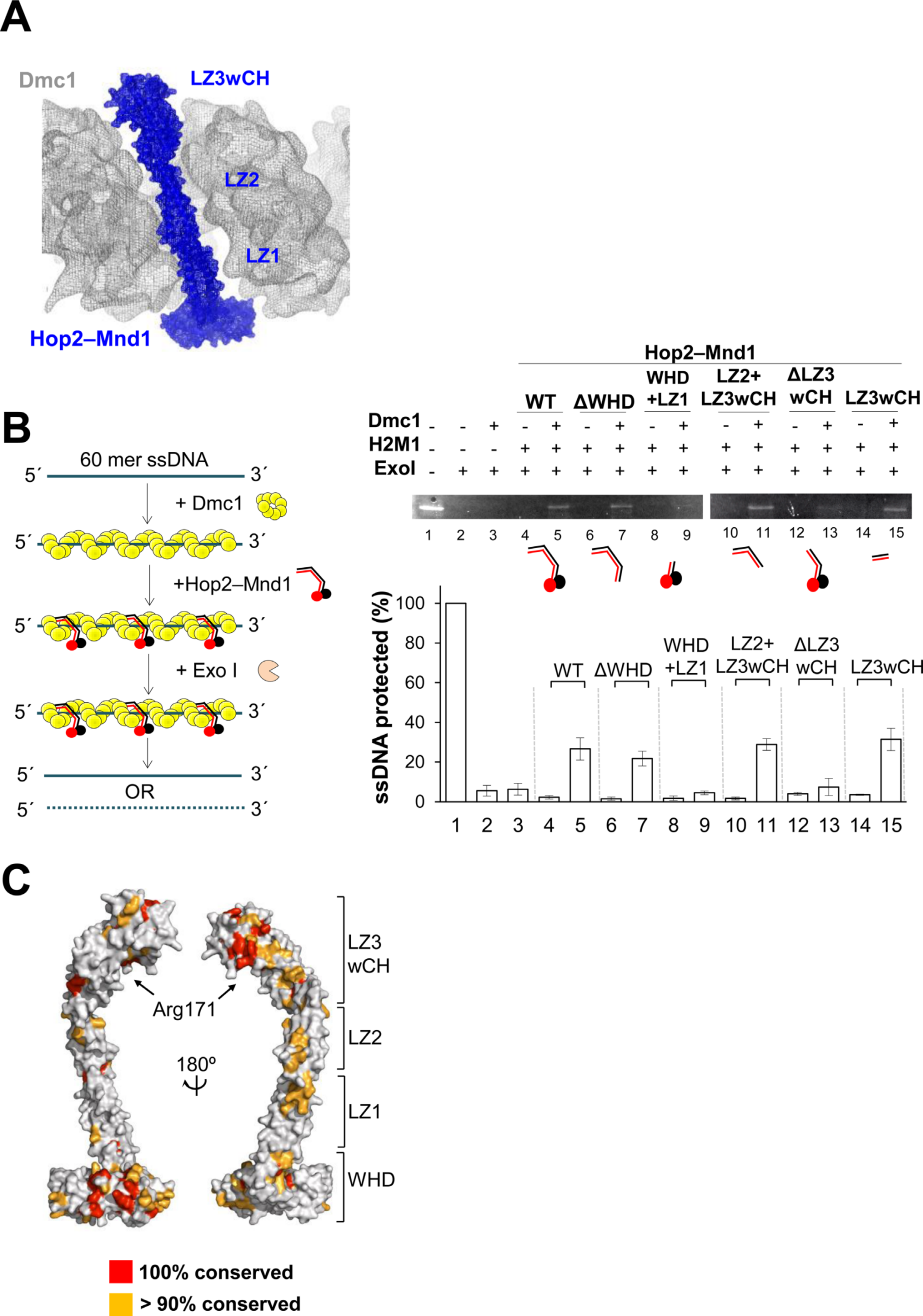
The C-terminal portion of Hop2–Mnd1 interacts with Dmc1 nucleofilament. (**A**) Fitting of the coiled coil of Hop2–Mnd1 into the helical groove of the Dmc1-ssDNA filament (EBI entry: EMD-1492). Surface of both Hop2–Mnd1 (blue) and human Dmc1-ssDNA filament (gray) are shown in mesh representation. (**B**) LZ3wCH of Hop2–Mnd1 is necessary for binding to Dmc1 nucleofilament. (*Left*) Schematic representation of the exonuclease I protection assay. (Right, top) Wild-type Hop2–Mnd1 and the indicated deletion mutants (2.5 μM) were individually incubated with Dmc1 nucleofilament and their ssDNA protection ability was analyzed by electrophoresis on a 15% native gel. (Right, bottom) The intensities of unreacted ssDNA relative to input ssDNA are shown. The experiment was performed in triplicate. (**C**) Mapping of conserved residues on the surface of Hop2–Mnd1.

In the Hop2–Mnd1 structure, the orientations of the three leucine zippers are different. Intriguingly, we found that the three leucine zippers in their respective orientations can be fitted into the helical groove in the filament of the Dmc1-ssDNA complex (Figure 6A) (44). While LZ1 and LZ2 could be snugly fitted into the groove, LZ3wCH having the capping helices appeared to cause some steric crash. In order to elaborate this observation, we produced five different deletion mutants and performed Exo I protection assays (Figure 6B). Cleavage of Dmc1-bound ssDNA by Exo I nuclease was suppressed by wild-type Hop2–Mnd1. Importantly, deletion constructs, which retain the LZ3wCH region (Figure 6B; ΔWHD, LZ2+LZ3wCH, LZ3wCH), exhibited a similar level of protection as wild type. In contrast, mutants lacking this region (Figure 6B; WHD+LZ1, ΔLZ3wCH) were far less protective. Thus, contrary to our expectation, the LZ3wCH region alone was sufficient for interacting with the Dmc1 nucleofilament. Induced-fit binding may be necessary for the interaction between LZ3wCH and the groove of the Dmc1 nucleofilament. Consistent with this notion, a proteolysis assay showed that mouse Hop2–Mnd1 complex affects the conformation of human Rad51 (45). Upon binding of LZ3wCH to the Dmc1 nucleofilament, LZ1 and LZ2 may passively position into the helical groove, and the flexibility of the LZ1–LZ2 and LZ2–LZ3 junctions may allow for their adjustments to the induced-fit conformational change of the Dmc1 nucleofilament.

The presented Hop2–Mnd1 structure with LZ3wCH as the binding motif explains the phenotype of a mutant allele of *Arabidopsis thaliana* HOP2 (hop2–3), which expresses a truncated Hop2 protein lacking residues 123–136 that retains the ability to bind to Dmc1 and DNA but forms less stable complexes with Mnd1 (46). These residues correspond to residues 127–136 of *G. lamblia* Hop2 that are located on LZ2, and thus unlikely to affect the structural integrity of LZ3wCH. Our observations also explain a recent finding that the flawed C-terminal 13 residue segment in the erroneously known open reading frame of yeast Hop2 had hampered the otherwise robust yeast Hop2–Mnd1 activity of stimulating Dmc1 (47).

Notably, mapping of the residues that are conserved in more than 90% of the top 100 hits in a *BLAST* search showed that conserved and surface-exposed residues are concentrated on LZ3wCH and the WHDs (Figure 6C), suggesting that these residues are likely to participate in conserved intermolecular interactions. One of these conserved residues, Arg171 of Hop2, is located in the middle of LZ3 and faces the Dmc1 nucleofilament in the model where the curved coiled coil of Hop2–Mnd1 spans the groove of the filament. Arg171 of *G. lamblia* Hop2 corresponds to Arg176 of mouse Hop2. Previously, R176A mutation in mouse Hop2 was shown to impair the presynaptic filament stabilization and D-loop formation by Hop2–Mnd1 (26). This mutational analysis is consistent with our deletion analysis and the model for interaction between Hop2–Mnd1 and the Dmc1 nucleofilament.

## DISCUSSION

While extensive studies have been conducted on Hop2–Mnd1, structural information at atomic level has been available only for the WHD of Hop2, hampering mechanistic understanding of this essential complex. We now present the crystal structure of full-length *G. lamblia* Hop2–Mnd1. Importantly, the structure explains a body of pre-existing information about Hop2–Mnd1 and provides new insights into the molecular function.

### Insights into the stimulatory role of Hop2–Mnd1 in Dmc1-mediated strand invasion

**Figure 7.**
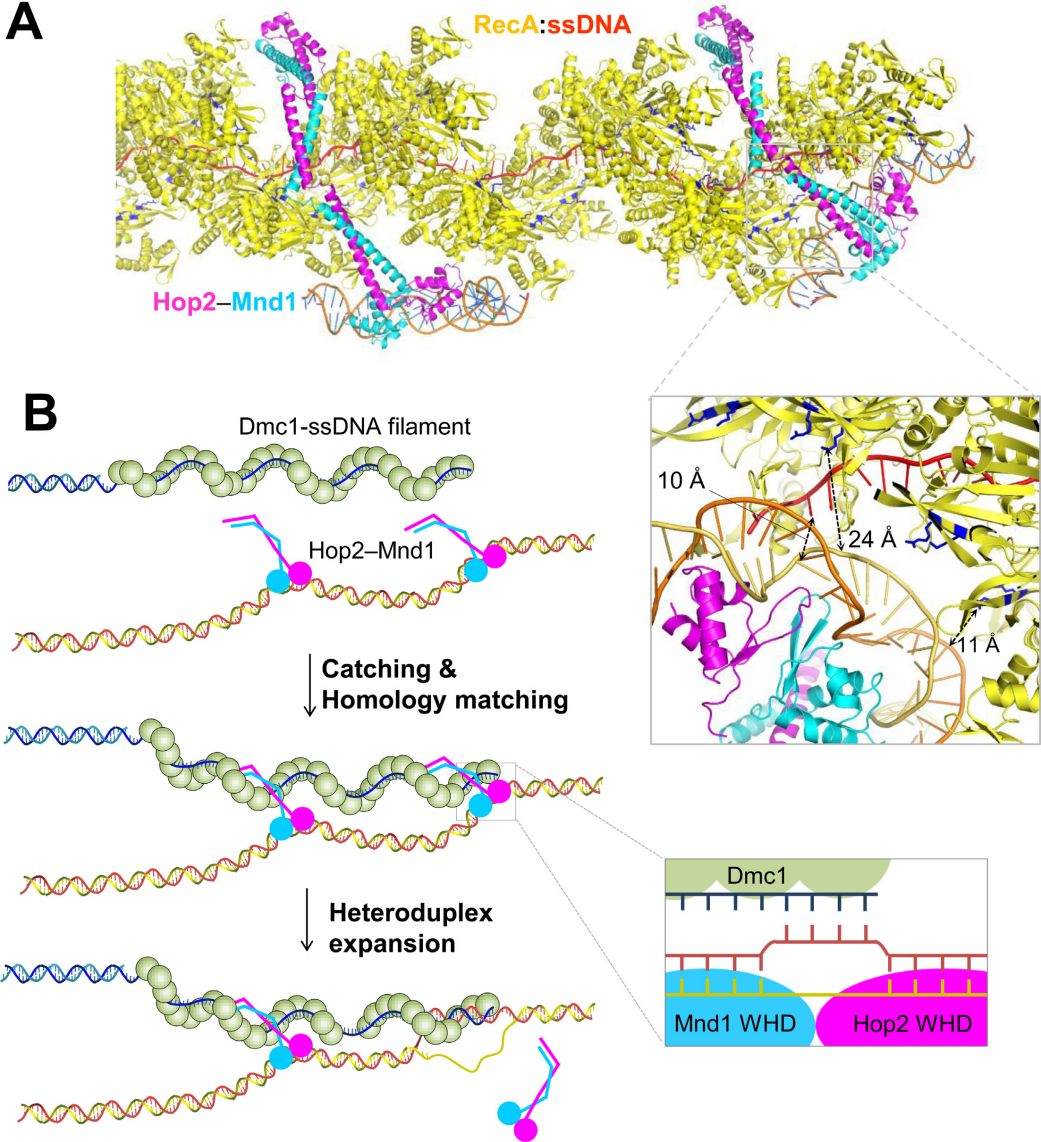
A model for Hop2–Mnd1-assisted strand invasion. (**A**) A model of Hop2–Mnd1 binding to RecA nucleofilament. The structural model obtained from the MD simulation was fitted into the groove of the crystal structure of RecA (yellow) bound to ssDNA (red) (PDB entry: 3cmu). The Site II residues are shown in sticks (blue), and representative distances are indicated. (**B**) A model for Hop2–Mnd1-assisted strand invasion. The Hop2–Mnd1 molecules catch the Dmc1 nucleofilament, which is then closely juxtaposed to (highly distorted) dsDNA bound to the WHD pair. If sequence matches most preferably at the end of the filament, DNA joint molecule is formed and strand invasion proceeds subsequently. This would require concomitant with Hop2–Mnd1 detachment from both dsDNA and the Dmc1 filament for the propagation of the heteroduplex.

Based on our analyses, we constructed a model for Hop2–Mnd1 bridging Dmc1 nucleofilament and recipient dsDNA. We employed the geometry-optimized structure of the WHDs of Hop2–Mnd1 bound to dsDNA and the atomic-resolution structure of the RecA-ssDNA complex, which is remarkably similar to the structure of human Dmc1 nucleofilament (44). Two Hop2–Mnd1 molecules were used to represent the binding of this complex at the end and in the middle of the filament. The coiled coil was placed into the helical groove, and the flexible WHD–LZ1 junction was adjusted to bring the WHDs close to ssDNA at the end of the filament or to avoid steric crash in the middle of the filament (Figure 7A).

At the end of the filament, the distorted portion of DNA bound to Hop2–Mnd1 is within 24 Å from the secondary DNA-binding sites on RecA and 10 Å apart from the end of ssDNA (Figure 7A). This binding mode reflects the dual-molecule experiments reported previously showing that the opening of the double helix of recipient DNA is required for recognition by the secondary DNA-binding site on RecA during homology sampling (48,49). The basic patch of the secondary DNA-binding site (Site II) of *E. coli* RecA is composed of Arg227, Arg243 and Lys245. These residues correspond to Arg124, Arg298 and Lys308 in *S. cerevisiae* Dmc1. A mutant Dmc1 bearing substitution of these residues with alanine (Dmc1-II3A) was previously shown to retain the filament forming but not D-loop forming activity as similarly observed for the Rad51-II3A mutant (15), indicating that the basic patches in Dmc1 and Rad51 are functionally similar to Site II of RecA.

According to the model, the WHD pair of Hop2–Mnd1 is too large to fit into the groove of the nucleofilament, unless substantial conformational change of the filament takes place. Therefore, Hop2–Mnd1-assisted homologous sequence comparison is likely to begin at the end of the filament to which the WHD pair can access without steric crash. This notion is in line with the observation that heteroduplex DNA formation proceeds with a preference to start at ssDNA ends on Rad51 nucleofilaments (50).

The proposed model implies that having two binding interfaces at the two distal ends of the curved structure is a key to bringing recipient distorted dsDNA close to Site II and ssDNA on the Dmc1 presynaptic filament, which would facilitate heteroduplex DNA formation between the recipient dsDNA and the Dmc1-bound ssDNA. Accordingly, we postulate a ‘catch and match’ model where Hop2–Mnd1 molecules prebound to chromatin DNA catch the Dmc1 nucleofilament and match homologous sequences between Dmc1-bound ssDNA and WHD-bound dsDNA. The initial base pairing, preferentially at the end of the filament, would promote further expansion of the heteroduplex on the target dsDNA (Figure 7B). Although the binding affinity between Hop2–Mnd1 and Dmc1 or Rad51 nucleofilament has not been documented, we suggest that it is weak based on published data (23,25,26) and our observations from a native gel-based protein binding assay employing high concentration of the proteins (not shown). Due to the low affinity, the Dmc1 nucleofilament would frequently dissociate from and reassociate with the Hop2–Mnd1 molecules that remain mostly bound to the target dsDNA owing to its high DNA-binding affinity (*K*_D_ of 100 nM). This on-and-off process may allow for homologous DNA search along the target DNA.

### Structural similarity with the Swi5–Sfr1 complex

**Figure 8.**
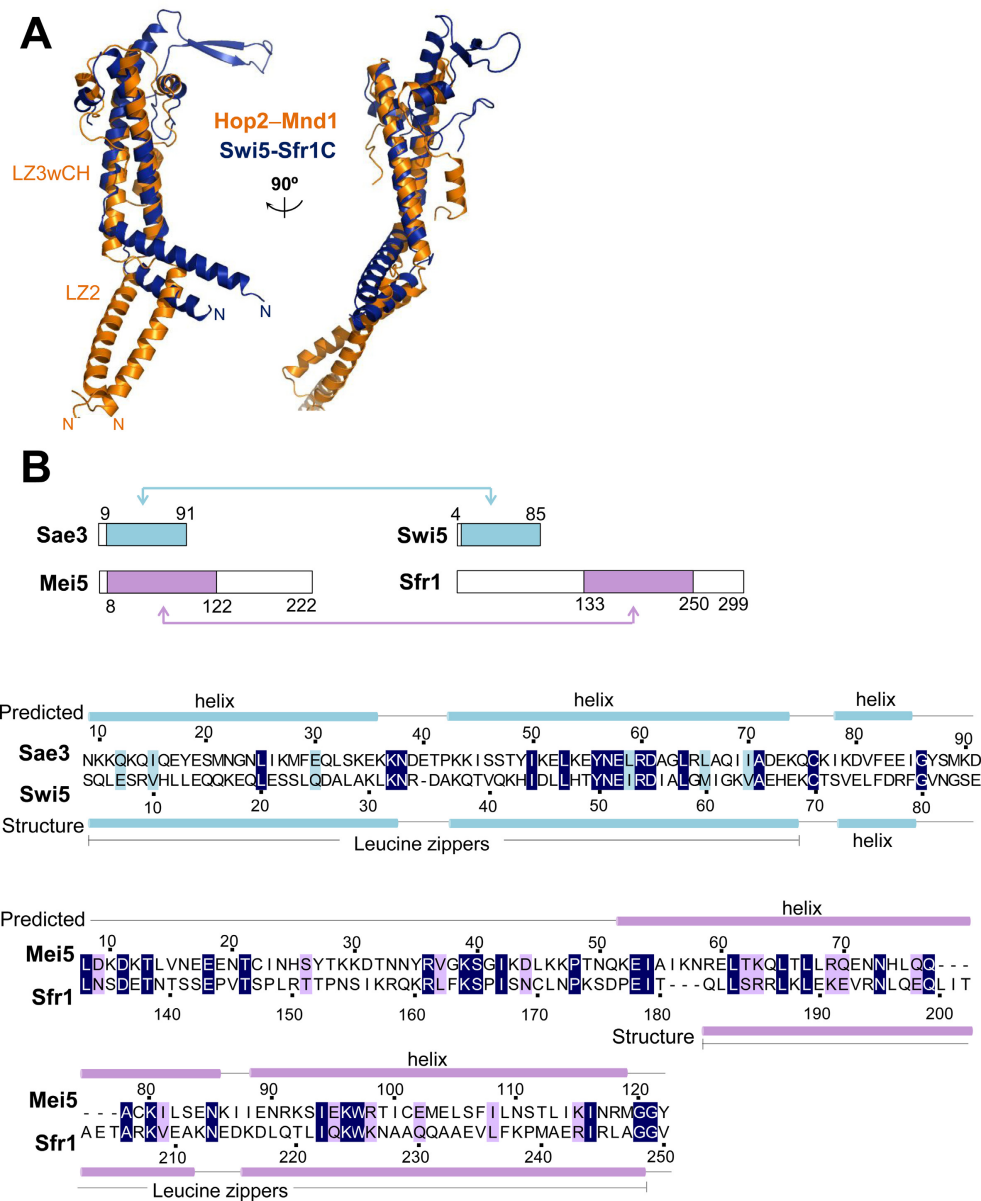
Structural and sequence similarity between Hop2–Mnd1, Swi5-Sfr1 and Mei5–Sae3. (**A**) Comparison between Hop2–Mnd1 and Swi5–Sfr1C. Structural superposition reveals close structural similarity, but with different directions of the kinks. The protruding β-sheet of Swi5-Sfr1C was shown to be flexible in solution and dispensable for stimulation of Rad51 (54). (**B**) Comparison between Swi5–Sfr1 and Mei5–Sae3. Schematic drawings of the four proteins. The colored boxes and arrows indicate the homologous regions, whose sequences are aligned below. These regions exhibit sequence identity of 31% between Sae3 and Swi5 and 21% between Mei5 and Sfr1. Predicted (for Sae3 and Mei5) or structure-based (for Swi5 and Sfr1) secondary structure elements are shown above and below the alignment, respectively.

According to protein sequence homology detection by HHpred (51) applied to the Protein Databank (PDB), both Hop2 and Mnd1 matched to the *Schizosaccharomyces pombe* mating-type switching protein Swi5, but not its binding partner protein Sfr1 (PDB entry: 3viq). Swi5 (85 residues) and Sfr1 (299 residues) are evolutionary conserved proteins and form a heterodimeric complex that stimulates Rad51- and Dmc1-mediated strand invasion (52). Intriguingly, a SAXS analysis of full-length yeast Swi5–Sfr1 revealed an extremely elongated dogleg-shaped structure (53), and the crystal structure of heterodimer between full-length Swi5 and a C-terminal 119 residue fragment of Sfr1 (Swi5–Sfr1C) forms a parallel coiled coil in a crescent shape, which comprises two leucine zippers with a kinked region in between (54). Furthermore, the second leucine zipper forms a helix bundle-like structure together with the C-terminal segments of Swi5 and Sfr1, exhibiting remarkable structural similarity with the LZ3wCH portion of Hop2–Mnd1 (Figure 8A). The elongated Swi5–Sfr1C heterodimer was previously shown to fit into the helical groove of a reconstituted model of Rad51 filament, and retains the essential function of the full-length Swi5–Sfr1 complex as an activator of Rad51 and Dmc1 via presynaptic filament stabilization (54,55). Thus, the uncovered structural similarity identifies the crescent-like leucine zippers in the two heterodimeric complexes as a common structural motif that interacts with the helical groove of the Dmc1 and/or Rad51 presynaptic filaments.

Notably, the N-terminal domain of Sfr1 exhibits dsDNA-binding affinity, and is necessary for the full activity of Swi5–Sfr1 in stimulating Rad51- and Dmc1-mediated strand-exchange reactions (54). Therefore, the N-terminal domain of Sfr1 might be functionally equivalent to the WHD pair in Hop2–Mnd1 despite completely different primary sequence between the two domains. The interaction between Swi5–Sfr1 and Dmc1 was suggested to be weak and transient as in the model we propose (54). Although further study is definitely required, the stimulation of Rad51- and Dmc1-mediated strand-exchange by the Hop2–Mnd1 and Swi5–Sfr1 heterodimers might be mechanistically similar.

*S. cerevisiae* Sae3 (91 residues) and Mei5 (222 residues) are homologs of Swi5 and Sfr1, respectively. They also form a heterodimer that physically interacts with Rad51, Dmc1 and replication protein A. Mei5–Sae3 preferentially binds to DNA fork structure and stimulates Dmc1-mediated D-loop formation together with Rad51 during meiosis (15,56–59). While the structure of Mei5–Sae3 is unavailable, it is likely to contain two leucine zippers as it exhibits sequence similarity to Swi5–Sfr1 (Figure 8B) and contains predicted coiled coil regions (not shown). Mei5–Sae3 might also interact with the ssDNA-binding proteins through the putative leucine zipper regions to stimulate their D-loop forming activity.

### Concluding remarks

In summary, full-length Hop2–Mnd1 is a curved rod-like structure with a WHD pair at one end and a helical bundle-like structure at the other distal end. This elongated structure, which is unexpectedly similar to the Swi5–Sfr1 structure, appears as a characteristic feature to bridge Dmc1 presynaptic filaments and dsDNA. The curved structure fits into the helical groove on Dmc1 nucleofilaments and this binding mode is presumed to bring recipient dsDNA close to the ssDNA and the secondary DNA binding sites on Dmc1 filament. Molecular simulations suggest that the WHDs juxtaposition via conserved intermolecular interactions might distort the recipient dsDNA, which would then facilitate homology sampling by the Dmc1-bound ssDNA.

It is largely unclear yet how the recombination mediators, including Hop2–Mnd1, function with the Dmc1 nucleofilament to catalyze homology search and synaptic filament formation that are enigmatically biased toward between homologs. The presented study provides an important framework for site-directed mutagenesis of Hop2–Mnd1 and Dmc1 in reconstituted *in vitro* systems and *in vivo* studies directed toward understanding of their molecular mechanisms.

## ACCESSION NUMBERS

The coordinates of the Hop2–Mnd1 structure together with the structure factors have been deposited in the Protein Data Bank with the PDB entry 4Y66.
